# Reduced antibody response to SARS-CoV-2 in COVID-19 patients with newly diagnosed diabetes: a retrospective observational study

**DOI:** 10.1186/s12902-023-01263-z

**Published:** 2023-01-06

**Authors:** Naoya Otsubo, Tatsuya Fukuda, Hiroko Beppu, Chisato Maki, Fumihiko Yasui, Tomohide Hanawa, Chise Sugita, Masanori Murakami, Tetsuya Yamada, Michinori Kohara, Sachiko Wakai

**Affiliations:** 1 Department of Endocrinology and Metabolism, Tokyo Metropolitan Health and Hospitals Corporation Okubo Hospital, 2-44-1 Kabuki-Cho, Shinjyuku-Ku, Tokyo, 160-8488 Japan; 2 Department of Molecular Endocrinology and Metabolism, Graduate School of Medical and Dental Sciences, Tokyo Medical and Dental University, Tokyo, Japan; 3 Department of Nephrology, Tokyo Metropolitan Health and Hospitals Corporation Okubo Hospital, Tokyo, Japan; 4 Department of Microbiology and Cell Biology, Tokyo Metropolitan Institute of Medical Science, Tokyo, Japan; 5 Department of Pulmonary Medicine, Tokyo Metropolitan Health and Hospitals Corporation Okubo Hospital, Tokyo, Japan

**Keywords:** Diabetes, Immune system, COVID-19, Antibodies, SARS-CoV-2

## Abstract

**Background:**

The coronavirus disease 2019 (COVID-19) pandemic has dramatically impacted global health, and patients with type 2 diabetes have been identified as a high-risk group for COVID-19 infection and the development of severe disease. In response, this study aimed to evaluate whether patients with type 2 diabetes infected with severe acute respiratory syndrome coronavirus 2 (SARS-CoV-2) could develop antibody responses in the same manner as patients without diabetes, and whether there is a difference in antibody response to SARS-CoV-2 between patients with diabetes diagnosed prior to hospitalization, and those with newly diagnosed diabetes.

**Methods:**

SARS-CoV-2-specific immunoglobulin G (IgG) levels were quantified using two iFlash 3000 Chemiluminescence Immunoassay analyzer kits (Shenzhen YHLO Biotech Co., Ltd.) to detect IgG antibodies specific for nucleocapsid protein (IgG-N), and specific for the S1 subunit of the spike protein (IgG-S1). In 124 hospitalized patients with COVID-19, 40 patients with type 2 diabetes were matched to 40 patients without diabetes using propensity score matching (PSM).

**Results:**

There was no difference in IgG-N and IgG-S1 levels between the patients with diabetes and those without. Of patients with diabetes, 31 patients had known diabetes and nine patients had newly diagnosed diabetes. The median levels of IgG-N at 7–13 days in patients with newly diagnosed diabetes were significantly lower than those in patients with known diabetes (IgG-N; 10.9 vs. 31.0 AU/mL, *p* = 0.031, IgG-S1; 7.5 vs. 24.4 AU/mL, *p* = 0.023).

**Conclusions:**

Even after adjusting for covariates using PSM, COVID-19 patients with type 2 diabetes had comparable antibody responses to patients without diabetes. Patients with newly diagnosed diabetes had lower IgG-N and IgG-S1 production in the second week of the disease compared with those with previously known diabetes.

## Introduction

The outbreak of coronavirus disease 2019 (COVID-19) caused by the severe acute respiratory syndrome coronavirus 2 (SARS-CoV-2) has become a global pandemic which the World Health Organization declared a public health emergency of international concern [1]. Compared with previously prevalent influenza strains, SARS-CoV-2 infection is clearly more severe [2], and COVID-19 has become one of the leading causes of death worldwide.

Patients with type 2 diabetes have been identified as a high-risk group for COVID-19 infection and the development of severe disease [3–7]. Patients with diabetes are thought to be more vulnerable to infectious disease because their humoral immune response and production of antigen-specific antibodies by B-lymphocytes are impaired [8]. A study in animals found that hyperglycemia impairs the function of immunoglobulin-producing B-lymphocytes [9], and some human studies have found that plasma immunoglobulin G (IgG) levels were lower in patients with diabetes [10, 11]. Previous research has also shown that after vaccination of diabetic patients, those with uncontrolled diabetes had impaired antibody response after influenza and hepatitis B vaccinations [12–14]. Similarly, the CAVEAT study found that virus-neutralizing antibody responses after COVID-19 vaccination were lower in diabetic patients with poor glycemic control compared with responses in participants without diabetes [15]. Given these findings, insufficient SARS-CoV-2 antibody production may partly explain the link between diabetes and poor clinical outcomes; however, little is known about the antibody response in COVID-19 patients with diabetes.

Undiagnosed diabetes patients are generally considered to have higher hemoglobin A1c (HbA1c) [16], and are reported to have a higher in-hospital mortality rate compared with those with a prior diagnosis of diabetes [17]. Therefore, undiagnosed diabetes could be associated with a poor prognosis for COVID-19. Indeed, a study reported that undiagnosed diabetes is a risk factor for severe COVID-19 [18]. Taking these factors into consideration, it is possible that patients with undiagnosed diabetes have an already particularly impaired antibody response at the time of admission for COVID-19; however, the detailed features of antibody response in patients with undiagnosed diabetes have to date not been determined.

This study retrospectively assessed whether patients with type 2 diabetes who were infected with SARS-CoV-2 could develop antibody responses similar to patients without diabetes. Additionally, whether patients with newly diagnosed diabetes have a different antibody response to SARS-CoV-2, compared with the response in patients with previously diagnosed diabetes upon admission was examined.

## Material and methods

### Study design and study population

Patients with type 2 diabetes who were admitted to Okubo Hospital, Japan, for the treatment of COVID-19 between 1 April 2020 and 28 March 2021 participated in this retrospective observational study. Patients aged over 20 years and who tested for anti-SARS-CoV-2 antibodies were included in the study. We excluded patients with an estimated glomerular filtration rate **(**eGFR) < 15 mL/min/1.73 m^2^　(all of whom were on hemodialysis). In addition, we excluded patients infected with human immunodeficiency virus (HIV) patients who had received organ transplants, and patients using immunosuppressing agent including tacrolimus and glucocorticoids before hospitalization. COVID-19 was diagnosed using a reverse transcription polymerase chain reaction test.

This study was carried out in accordance with the Declaration of Helsinki. The protocol for this study was approved by the Okubo Hospital’s ethical review committee (No. 2020–11). Written informed consent was not required because of the study’s retrospective design. An outline of the analysis was displayed on the hospital website, which gave patients the option to opt out of the study.

### Antibody measurement

**Fig. 1:**
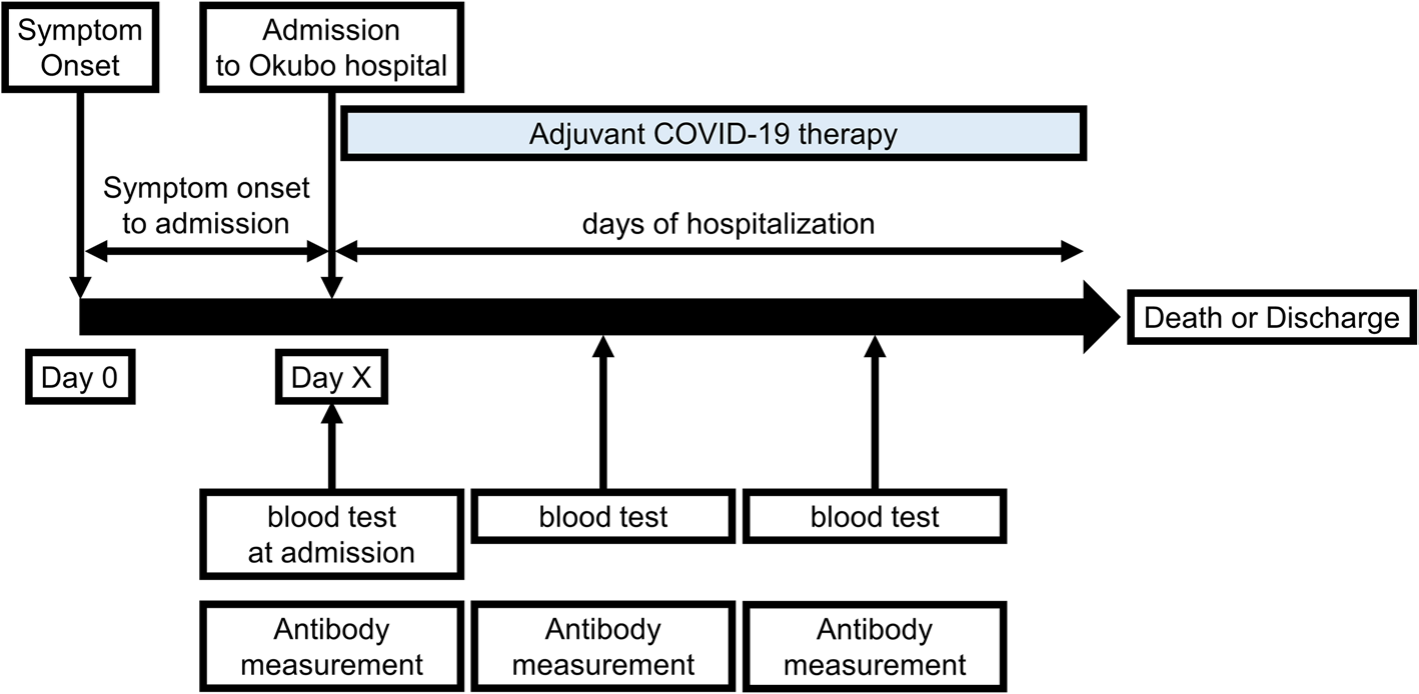
The time course of antibody measurement and in-hospital treatment for COVID-19

Figure 1 shows the time course of antibody measurement for COVID-19. Blood samples were taken on the day of admission and every few days thereafter, and the remaining serum was frozen and stored to measure antibodies. Antibody measurements were performed on all remaining sera from blood samples taken during hospitalization. Measurement of concentrations of anti-SARS-CoV-2 antibodies using an iFlash 3000 chemiluminescence immunoassay analyzer (Shenzhen YHLO Biotech Co., Ltd. China). Two kits were utilized; the iFlash-SARS-CoV-2 IgG-S1 kit, and the iFlash-SARS-CoV-2 IgG kit. The iFlash-SARS-CoV-2 IgG-S1 kit detected immunoglobulin G specific to the S1 subunit of the spike protein (IgG-S1). Although the iFlash-SARS-CoV-2 IgG kit used a combination of both N and S antigens, IgG levels measured by this kit were reported to be strongly correlated with antibody levels against the N protein of SARS-CoV-2 [19]. Therefore, the iFlash-SARS-CoV-2 IgG kit was utilized to detect IgG specific to the N protein (IgG-N). In accordance with the manufacturer’s instructions, results with values ≥ 10 arbitrary units (AU)/mL were considered positive.

### Clinical data collection

Patient data, including age, gender, body mass index (BMI), smoking status, history of COVID-19 vaccination, comorbidities including hypertension, chronic obstructive pulmonary disease (COPD), cardiovascular disease (CVD), and cancer history, laboratory test results, and clinical outcomes were extracted from patients’ electronic medical records. Hypertension was diagnosed based on medical record history or the current use of antihypertensive medications. The presence of diabetes at admission was classified into the following two categories. 1) Known diabetes was defined based on either a self-reported history of diabetes, medical records prior to admission, reported diagnosis of diabetes, or treatment with glucose-lowering medications. 2) Newly diagnosed diabetes was defined as the presence of HbA1c ≥ 6.5% plus one of the following states in patients who reported that they had never been previously diagnosed with diabetes mellitus; fasting plasma glucose ≥ 126 mg/dL, or random blood glucose ≥ 200 mg/dL. COPD was diagnosed based on medical record history, or the presence of typical COPD changes seen in high-resolution computed tomography scans. CVD was diagnosed based on a medical record history of stroke, unstable angina, myocardial infarction, percutaneous coronary intervention, coronary bypass grafting, angioplasty, or major amputation because of peripheral arterial disease.

At the time of admission, the 4C mortality score was calculated in each patients with diabetes to predict in-hospital mortality [20].

The severity of COVID-19 was determined based on each patient’s worst condition during hospitalization, and was classified into the following three categories: mild or moderate, severe, or critical, according to the COVID-19 Treatment Guidelines Panel of the United States National Institutes of Health [21]. Critical disease was defined as requiring non-invasive positive pressure ventilation or invasive ventilation during hospitalization, or in cases in which the patient died from COVID-19. In patients without critical illness, severe disease was defined as having a partial pressure of oxygen/fraction of inspired oxygen (P/F) ratio < 300, or having an oxygen saturation level (SpO2) < 94% during hospitalization. Symptomatic COVID-19 that was neither critical nor constituted severe illness was defined as mild or moderate illness.

All COVID-19 patients with type 2 diabetes had their blood glucose levels monitored and received hypoglycemic therapy in the Okubo Hospital from three diabetologists certified by the Japan Diabetes Society. Every day, blood glucose measurements were taken before each meal in patients with diabetes utilizing the Glutest Neo Alpha kit (Sanwa Kagaku Kenkyusho Co., Ltd. Japan). In some patients, blood glucose level was measured at around 10 pm, in addition to the three mealtime measurements. In contrast, patients whose blood glucose levels were unlikely to rise above 200 mg/dl had their blood glucose level measured once a day before meals. The daily blood glucose level for each patient was defined as the average of all measured blood glucose values in a day. Patients which had an average value of daily blood glucose levels > 200 mg/dL during hospitalization within 13 days of COVID-19 onset were defined as experiencing consistent hyperglycemia.

### Statistical analysis

**Fig. 2:**
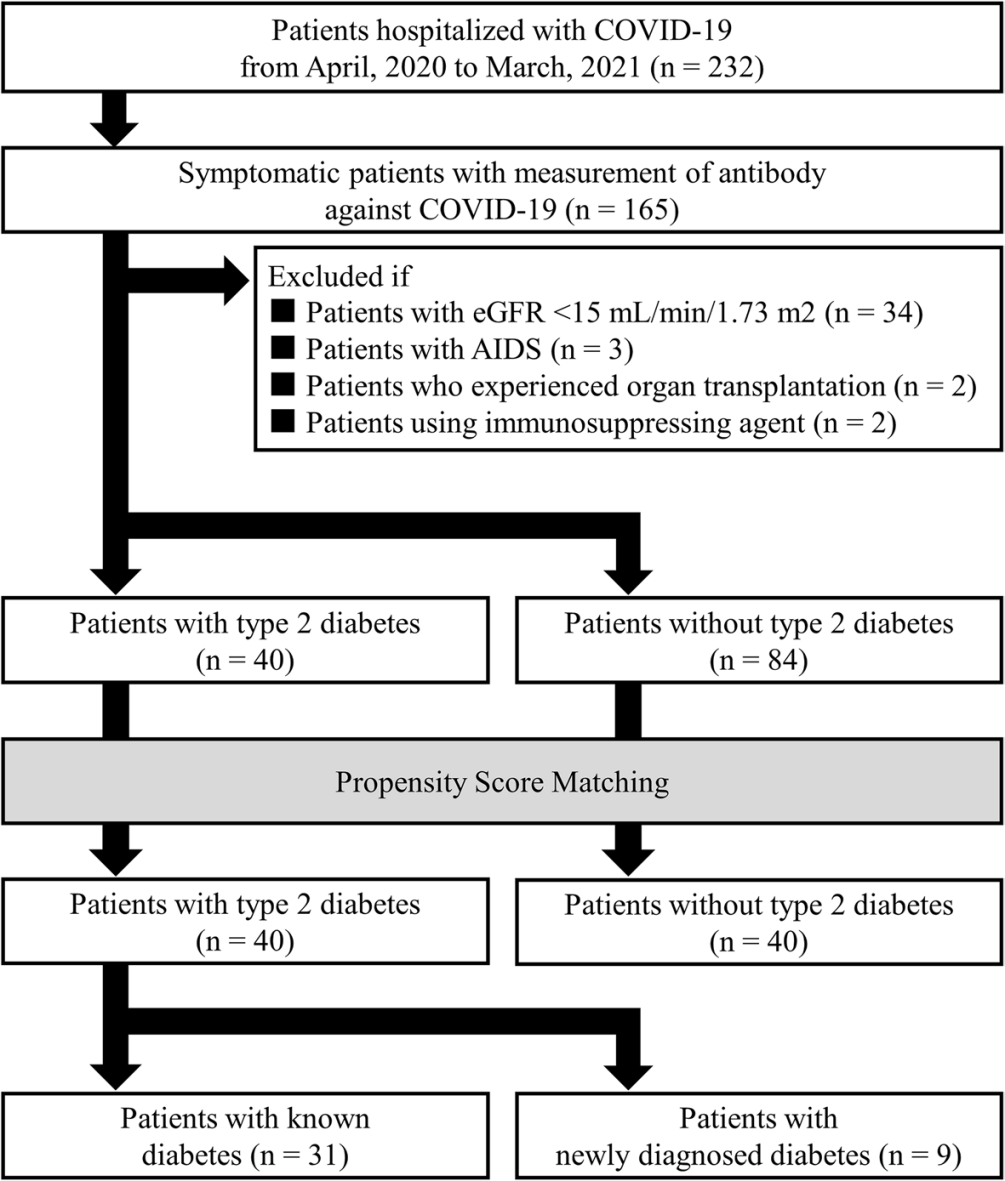
Study flow chart

According to the data distribution, data were presented as the mean ± standard deviation, median with interquartile range (IQR), or percentage. The chi-squared test or Fisher’s exact test was used to compare categorical variables. The *t*-test or Mann–Whitney *U* test was used to compare quantitative variables, depending on the data distribution. Each patient’s propensity score for type 2 diabetes was calculated using a logistic regression model that included covariates (age, gender, BMI, hypertension, CVD, and smoking status) to balance covariate distribution between patients with and without diabetes. Then, the propensity scores were used to perform 1:1 matching between patients with type 2 diabetes and those without type 2 diabetes using the nearest neighbor algorithm. Finally, 80 patients were selected for the propensity‐matched population (Fig. 2). *P* values < 0.05 were considered statistically significant. Statistical analyses were performed using SPSS v.21.0 (IBM Corp., Armonk, NY, USA).

## Results

### Clinical characteristics

Study flow chart are shown in Fig. 2. Of the 165 patients with symptomatic COVID-19 who were hospitalized and received antibody measurement during the study period, we excluded the 34 patients with an eGFR < 15 mL/min/1.73 m^2^, 3 patients infected with HIV, 2 patients who had received organ transplants, and 2 patients using immunosuppressing agent. Consequently, 124 patients meeting these criteria were selected from the overall patient population (Fig. 2).

The clinical characteristics of the overall population and the propensity score-matched population are shown in Table 1. Propensity scores were used to match 40 patients with type 2 diabetes with 40 patients who did not have type 2 diabetes. The patients’ ages, BMI scores, gender and smoker proportions, and incidence of comorbidities including hypertension, COPD, CVD, and cancer, were similar in the two propensity score-matched populations. All the patients included in the study did not receive the COVID-19 vaccine. All laboratory parameters upon admission, including C-reactive protein (CRP) (patients with type 2 diabetes: 4.5 mg/dL vs. patients without type 2 diabetes: 3.6 mg/dL, *p* = 0.441), and ferritin (patients with type 2 diabetes: 491 ng/mL vs. patients without type 2 diabetes: 511 ng/mL, *p* = 0.252), were comparable between the patients with and without type 2 diabetes, but HbA1c levels were found to differ more significantly (patients with type 2 diabetes: 7.4% vs. patients without type 2 diabetes: 5.9%, *p* < 0.001). The proportions of patients with type 2 diabetes who received steroids, favipiravir, and remdesivir were comparable to usage rates seen among patients without diabetes. The severity of COVID-19 and mortality rate were also comparable between patients with and without type 2 diabetes (patients with type 2 diabetes: 10% vs. patients without type 2 diabetes: 8%, *p* = 0.692).

**Table 1:** Baseline clinical characteristics, comorbidities, and the clinical outcomes

	**Overall population**			**Propensity score matched population**		
	**Patients with diabetes**	**Patients without diabetes**	***p* value**	**Patients with diabetes**	**Patients without diabetes**	***p* value**
	*n* = 40	*n* = 84		*n* = 40	*n* = 40	
Age, yr	69.0 ± 15.4	56.7 ± 23.7	0.003	69.0 ± 15.4	69.4 ± 20.1	0.931
Female sex	11 (28)	29 (35)	0.434	11 (28)	14 (35)	0.630
Body mass index, kg/m^2^	24.5 ± 3.8	23.4 ± 3.7 (n = 78)	0.203	24.5 ± 3.8	23.7 ± 4.2	0.400
Current Smoker	16 (40)	38 (45)	0.661	16 (40)	17 (43)	0.644
Symptom onset to admission, d	6.8 ± 3.7	5.9 ± 3.4	0.203	6.8 ± 3.7	6.0 ± 4.0	0.382
**Comorbidities**						
Hypertension	24 (60)	26 (31)	0.124	24 (60)	20 (50)	0.369
COPD	3 (8)	4 (5)	0.537	3 (8)	2 (5)	0.642
Cardiovascular disease	10 (25)	4 (5)	0.001	10 (25)	5 (13)	0.252
Cancer history	4 (10)	8 (10)	0.933	4 (10)	4 (10)	1.000
**Laboratory parameters at admission**						
HbA1c, %	7.4 (7.0–8.1)	5.9 (5.6–6.1)	< 0.001	7.4 (7.0–8.1)	5.9 (5.7–6.2)	< 0.001
White blood cells, 10^9^/L	5.4 (4.3–6.6)	5.0 (4.0–6.2)	0.483	5.4 (4.3–6.6)	5.1 (4.1–6.5)	0.465
Neutrophils, 10^9^/L	3.2 (2.7–4.7)	3.0 (2.4–4.1)	0.446	3.2 (2.7–4.7)	3.2 (2.6–4.2)	0.609
Lymphocyte, 10^9^/L	1.1 (0.7–1.3)	1.0 (0.7–1.4)	0.784	1.1 (0.7–1.3)	1.0 (0.7–1.3)	0.744
Albumin, g/dL	3.6 (3.1–3.9)	3.9 (3.4–4.3)	0.022	3.6 (3.1–3.9)	3.7 (3.1–4.2)	0.620
BUN, mg/dL	15.5 (12.5–23.8)	11.8 (9.6–16.5)	0.008	15.5 (12.5–23.8)	13.1 (10.4–20.9)	0.178
Serum creatinine, mg/dL	0.80 (0.70–0.96)	0.81 (0.66–1.02)	0.984	0.80 (0.70–0.96)	0.81 (0.67–1.07)	0.931
eGFR, mL/min per 1.73 m^2^	67.9 (55.6–81.5)	72.4 (57.8–90.0)	0.149	67.9 (55.6–81.5)	65.5 (50.9–82.9)	0.859
C-reactive protein, mg/dL	4.5 (1.4–8.4)	3.25 (0.75–5.77)	0.212	4.5 (1.4–8.4)	3.6 (1.1–7.1)	0.441
Ferritin, ng/mL	491 (279–1001)	302 (209–584)	0.048	491 (279–1001)	511 (226–666)	0.252
D-dimer, µg/mL	0.95 (0.78–1.77)	0.90 (0.68–1.24)	0.261	0.95 (0.78–1.77)	0.99 (0.75–1.55)	0.893
**Adjuvant COVID-19 therapy**						
Steroids	23 (58)	33 (39)	0.057	23 (58)	20 (50)	0.501
Remdesivir	11 (28)	10 (12)	0.030	11 (28)	5 (13)	0.162
Favipiravir	31 (78)	55 (65)	0.174	31 (78)	30 (75)	0.792
**Severity**						
Mild or Moderate	27 (68)	69 (82)	0.068	27 (68)	29 (73)	0.625
Severe	7 (18)	8 (10)	0.203	7 (18)	4 (10)	0.330
Critical	6 (15)	7 (8)	0.257	6 (15)	7 (18)	0.761
**Clinical outcomes**						
days of hospitalization	14.9 ± 10.7	11.9 ± 5.5	0.124	14.9 ± 10.7	13.4 ± 6.36	0.396
Death	4 (10)	3 (4)	0.147	4 (10)	3 (8)	0.692

Data are expressed as mean ± standard deviation (SD), median [interquartile range (IQR)], or as number [proportion (%)]

Abbreviations: COPD chronic obstructive pulmonary disease, BUN blood urea nitrogen, eGFR estimated glomerular filtration ratio, COVID-19 coronavirus disease 2019

*P* value for difference between groups in percent (Chi-square test or Fisher’s exact test), means (t test), or medians (Mann–Whitney U test)

### Antibody response to SARS-CoV-2 in patients with type 2 diabetes and those without

**Fig. 3:**
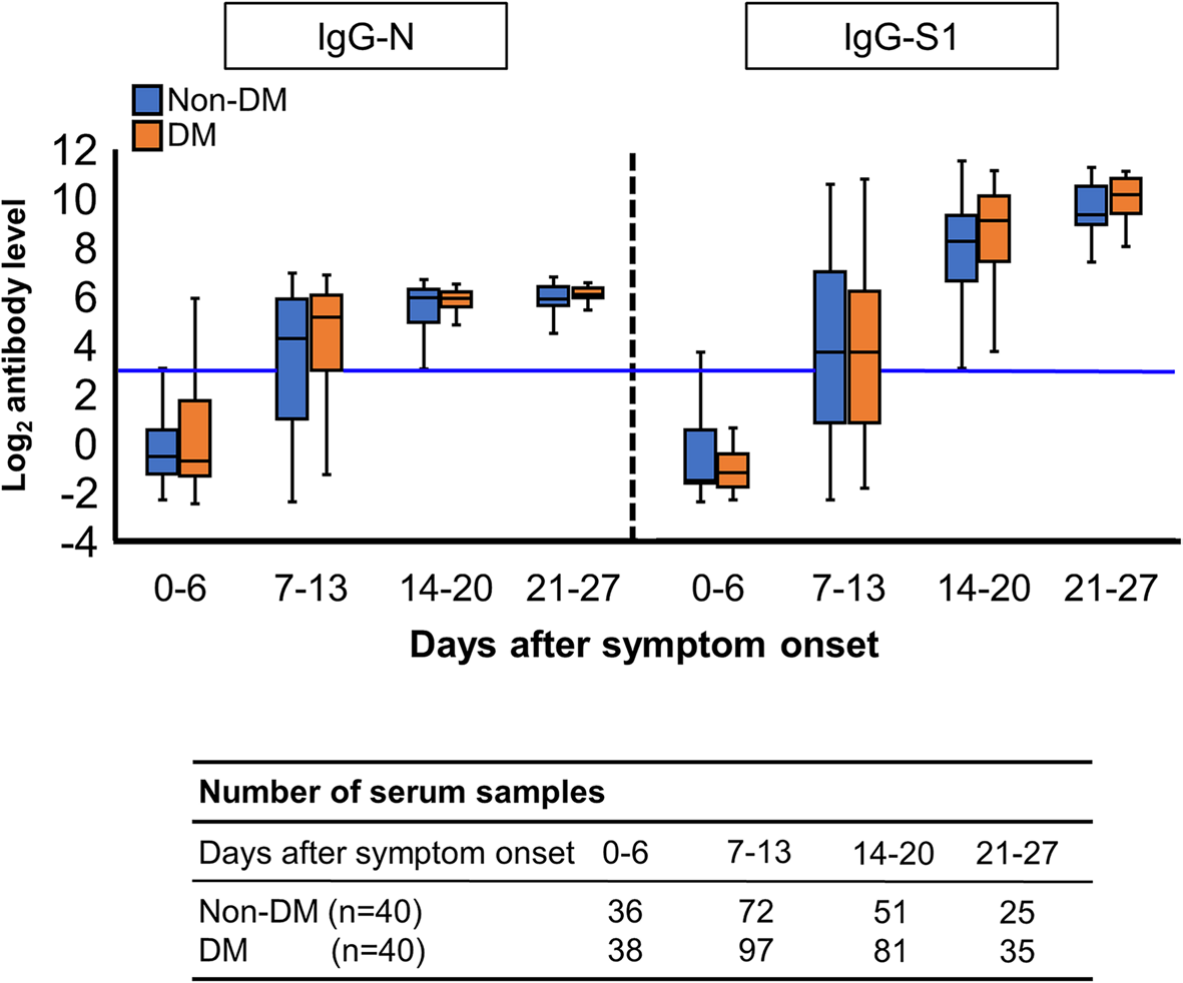
Kinetics of anti-SARS-CoV-2 IgG-N and IgG-S1 in 40 patients with type 2 diabetes and 40 patients without diabetes Antibody levels were log2-transformed. Blue lines represent the threshold value for positive. Comparisons of antibody titers in the same time period were performed using the Mann–Whitney U-test. *P < 0.05. Abbreviations: DM, diabetes mellitus; IgG-N, immunoglobulin G specific for nucleocapsid protein; IgG-S1, immunoglobulin G specific for the S1 subunit of the spike protein; SARS-CoV-2, severe acute respiratory syndrome coronavirus 2

The kinetics of anti-SARS-CoV-2 antibodies in 40 patients with type 2 diabetes and in 40 patients without type 2 diabetes are shown in Fig. 3. We measured IgG-S1 and IgG-N concentrations in 435 samples collected from 80 propensity score-matched patients up to 27 days after the onset of symptoms. The median number of antibody measurements performed during hospitalization did not differ significantly between the two groups (patients with type 2 diabetes: six measurements vs. patients without type 2 diabetes: five measurements, *p* = 0.100). The number of patients with confirmed IgG-N seropositivity at 14–20 days were 26 of 27 (96%) and 19 of 21 (90%) in patients without diabetes, and in those with diabetes, respectively. The number of patients with confirmed IgG-S1 seropositivity at 14–20 days were 27 of 27 (100%) and 21 of 21 (100%) in patients without diabetes, and in those with diabetes, respectively. For all time periods up to day 27 after COVID-19 onset, there were no differences observed in IgG-S1 and IgG-N levels between the patients with diabetes and those without diabetes.

### Comparison of antibody responses to SARS-CoV-2 between the patients with known diabetes and those with newly diagnosed diabetes

Among patients with type 2 diabetes in the propensity score-matched population, nine patients were newly diagnosed with diabetes upon admission. Table 2 shows the clinical characteristics of the patients with known diabetes and those with newly diagnosed diabetes. The ages, BMIs, proportions of females and current smokers, and comorbidities, were similar in the propensity score-matched population. All laboratory parameters upon admission, including HbA1c (patients with known diabetes: 7.4% vs. patients with newly diagnosed diabetes: 7.4%, *p* = 0.795), CRP (patients with known diabetes: 4.5 mg/dL vs. patients with newly diagnosed diabetes: 5.9 mg/dL, *p* = 0.388), and ferritin (patients with known diabetes: 385 ng/mL vs. patients with newly diagnosed diabetes: 883 ng/mL, *p* = 0.130) were comparable between the patients with known diabetes and the patients with newly diagnosed diabetes. The proportions of patients with known diabetes who received steroids, favipiravir, and remdesivir were comparable with those in patients with newly diagnosed diabetes. The severity of COVID-19 and the mortality rates between patients with known diabetes and patients with newly diagnosed diabetes were comparable.

**Table 2:** Baseline clinical characteristics, comorbidities, and the clinical outcomes in patients with known diabetes and newly diagnosed diabetes

	**Known diabetes**	**Newly diagnosed diabetes**	***p* value**
	*n* = 31	*n* = 9	
Age, yr	71.8 ± 14.4	61.0 ± 16.6	0.056
Female sex	9 (30)	2 (22)	0.667
Body mass index, kg/m^2^	24.4 ± 3.7	25.0 ± 4.3	0.642
Current Smoker	12 (38)	4 (44)	0.693
Symptom onset to admission, d	6.7 ± 3.7	7.5 ± 3.8	0.598
**Comorbidities**			
Hypertension	23 (74)	1 (11)	0.003
COPD	2 (7)	1 (11)	0.728
Cardiovascular disease	10 (32)	0 (0)	0.086
Cancer history	2 (7)	2 (22)	0.540
**Laboratory parameters at admission**			
HbA1c, %	7.4 (7.0–8.0)	7.4 (6.8–8.8)	0.795
White blood cells, 10^9^/L	5.9 (4.2–6.7)	5.0 (4.3–5.1)	0.136
Neutrophils, 10^9^/L	3.9 (2.4–4.9)	3.2 (2.9–3.3)	0.566
Lymphocyte, 10^9^/L	1.1 (0.7–1.3)	1.1 (0.8–1.3)	0.769
Albumin, g/dL	3.6 (2.9–3.9)	3.8 (3.5–4.0)	0.183
BUN, mg/dL	17.0 (12.9–28.5)	12.5(10.7–15.4)	0.068
Serum creatinine, mg/dL	0.81 (0.76–0.99)	0.74 (0.60–0.83)	0.095
eGFR, mL/min per 1.73 m^2^	62.6 (53.1–73.8)	80.7 (70.6–88.5)	0.056
C-reactive protein, mg/dL	4.5 (1.3–7.0)	5.9 (3.3–12.4)	0.388
Ferritin, ng/mL	385 (274–874)	883 (733–1509)	0.130
D-dimer, µg/mL	1.06 (0.84–1.91)	0.76 (0.67–0.96)	0.126
The 4C mortality score	11 (7–14)	10 (8–10)	0.269
**Adjuvant COVID-19 therapy**			
Steroids	17 (55)	6 (67)	0.527
Remdesivir	8 (26)	3 (33)	0.656
Favipiravir	24 (77)	7 (78)	0.827
**Severity**			
Mild or Moderate	22 (71)	5 (56)	0.499
Severe	6 (19)	1 (11)	0.496
Critical	3 (10)	3 (33)	0.156
**Clinical outcomes**			
days of hospitalization, d	16.7 ± 11.4	10.7 ± 7.3	0.248
Death	4 (13)	0 (23)	0.557

Data are expressed as mean ± standard deviation (SD), median [interquartile range (IQR)], or as number [proportion (%)]

Abbreviations: *COPD* Chronic obstructive pulmonary disease, *BUN* Blood urea nitrogen, *eGFR* Estimated glomerular filtration ratio, *COVID-19* Coronavirus disease 2019

*P* value for difference between groups in percent (Chi-square test or Fisher’s exact test), means (t test), or medians (Mann–Whitney U test)

**Fig. 4:**
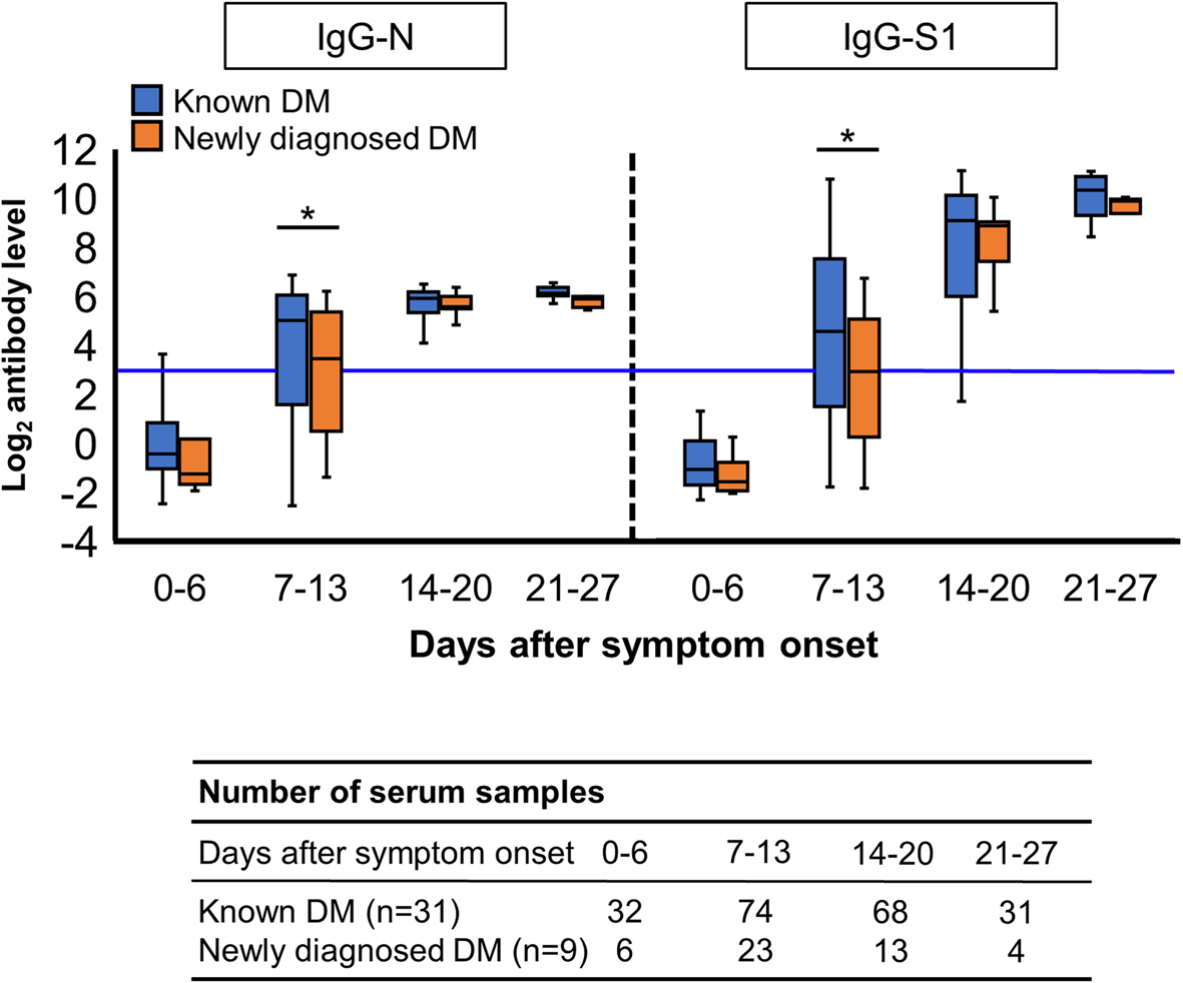
Kinetics of anti-SARS-CoV-2 IgG-N and IgG-S1 in 31 patients with known diabetes and 9 patients with newly diagnosed diabetes Antibody levels were log2-transformed. Blue lines represent the threshold value for positive. Comparisons of antibody titers in the same time period were performed using the Mann–Whitney U-test. *P < 0.05.

The kinetics of anti-SARS-CoV-2 antibodies in 31 patients with known diabetes and nine patients with newly diagnosed diabetes are shown in Fig. 4. The median (IQR) levels of IgG-N during days 7–13 in patients with newly diagnosed diabetes were significantly lower than those in patients with known diabetes (10.9 [1.6–38.2] vs. 31.0 [2.9–66.2] AU/mL, *p* = 0.031), and the median (IQR) levels of IgG-S1 during days 7–13 in patients with newly diagnosed diabetes were significantly lower than those in patients with known diabetes (7.5 [1.2–33.7] vs. 24.4 [2.8–185.0] AU/mL, *p* = 0.023).

**Fig. 5:**
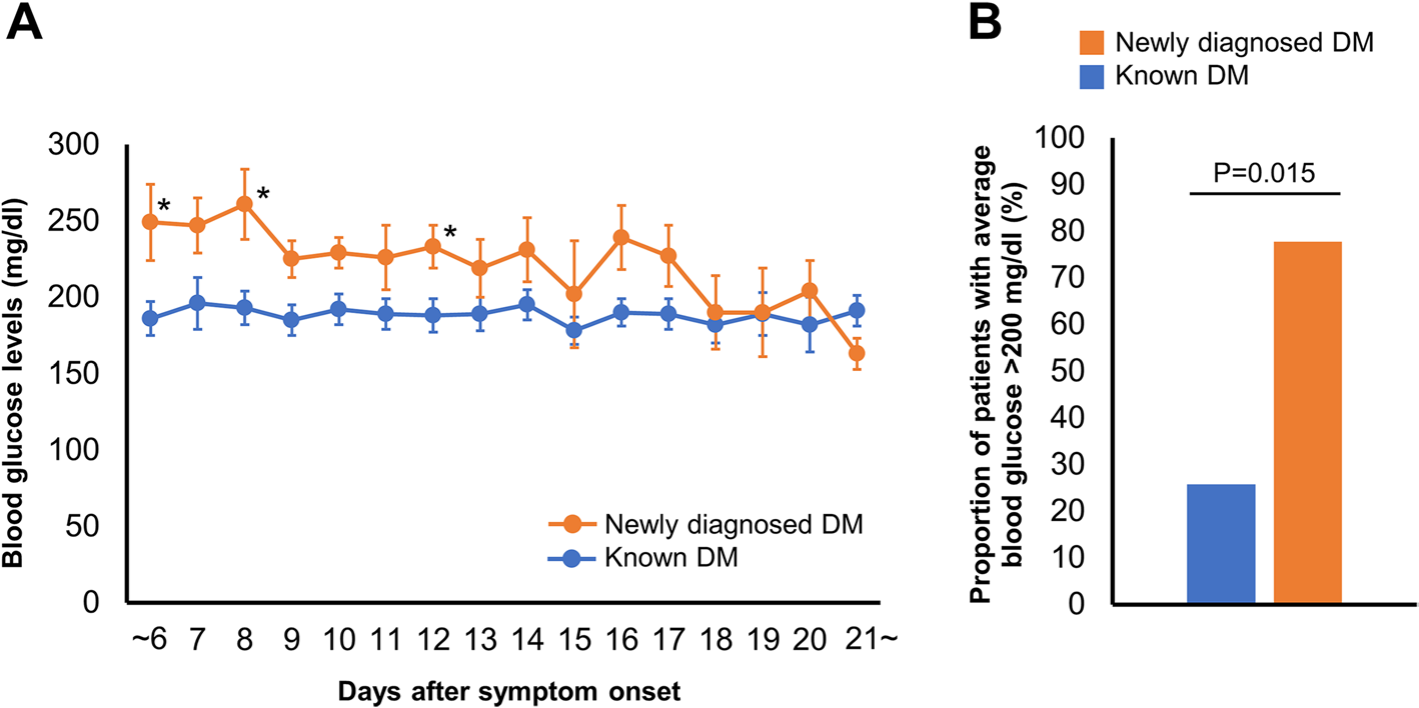
Glycemic control in hospitalized patients within 2 weeks of COVID-19 onset blood glucose levels up to day 6 and daily blood glucose levels from days 7 to 13 after COVID-19 onset in patients with known diabetes (blue) and newly diagnosed diabetes (orange). Data are presented as means ± SEM. *P < 0.05 at individual time points. the proportion of patients who experienced sustained hyperglycemia which is defined as an average value of daily blood glucose levels > 200 mg/dl during hospitalization within 13 days of COVID-19 onset in patients with known diabetes (blue) and newly diagnosed diabetes (orange).Abbreviations: DM, diabetes mellitus.

Blood glucose levels upon admission were investigated next and are shown in Fig. 5 A. Blood glucose levels were found to be higher in patients with newly diagnosed diabetes compared with levels seen in patients with known diabetes. This was seen up to six days from onset, and on days 8 and 12. Figure 5 B shows the proportion of patients who experienced sustained hyperglycemia, which was defined as an average daily blood glucose level > 200 mg/dL during hospitalization within 13 days of COVID-19 onset in patients with either known diabetes or newly diagnosed diabetes. The proportion of patients with sustained hyperglycemia was significantly higher in patients with newly diagnosed diabetes than in patients with known diabetes.

## Discussion

This study found that patients with diabetes had similar SARS-CoV-2 antibody responses compared with those without diabetes. At three weeks after symptom onset, the majority of patients with COVID-19, regardless of diabetes, had developed specific antibodies, including IgG-S1 and IgG-N, which is consistent with findings of a recent study [22].

The possibility of an inadequate humoral immune response in patients with diabetes had been reported in a previous study [8], and another study of 31 COVID-19 patients revealed the possibility that patients with diabetes may not be able to produce sufficient antibodies against SARS-CoV-2 [23]. In contrast, Lampasona V et al. reported that patients with diabetes can develop IgG against SARS-CoV-2 comparable to patients without diabetes [24]. The present study demonstrated that patients with diabetes can develop IgG antibodies similar to patients without diabetes, which is consistent with the results of Lampasona V et al. The inconsistency among these findings might be due to various complication characteristics in patients with diabetes. For example, it has been reported that patients with cardiovascular disease have a weak antibody response after COVID-19 vaccination [25]. Additionally, antibody responses were previously demonstrated to be delayed in patients undergoing hemodialysis [26]. Therefore, the presence or absence of such diabetes-related complications, including cardiovascular disease or renal disease, may be the determining factor in antibody response to SARS-CoV-2.

We further examined the characteristics of patients with diabetes who had impaired antibody response, and found that patients with newly diagnosed diabetes had lower levels of IgG-S1 during the second week of COVID-19 compared with patients with known diabetes. Anti-spike IgG antibodies have been found to be significantly correlated with virus-neutralizing antibody titers [27]. This implies that they play an important role in protection against SARS-CoV-2 infection. Indeed, recent studies have demonstrated that patients who died from COVID-19 had delayed anti-spike IgG production and neutralizing antibody response in the second week after symptom onset compared to levels seen in discharged patients [28, 29]. Given these findings, our data suggest that patients with newly diagnosed diabetes may be associated with a poor prognosis of COVID-19 due to the delayed production of anti-spike IgG.

It has been reported that while providing inpatient care for COVID-19 patients, that those with newly diagnosed diabetes have been seen to have more difficulty in managing glycemic control during the first week of hospitalization compared with patients with pre-existing diabetes [30]. Consistent with a previous study, we also found patients with newly diagnosed diabetes had higher blood glucose levels than those with previously diagnosed diabetes up to the second week of COVID-19 onset. Given these findings, poor glycemic control within two weeks of COVID-19 onset may be one of the causes of delayed antibody response, which presumably contributes to a poor COVID-19 prognosis. Although the present study did not find differences in severity or mortality rate between the patients with newly diagnosed diabetes and known diabetes, this may be due to the fact that the patients with known diabetes tended to be older and tended to have more cardiovascular disease, both of which are known risk factors for COVID-19 [31, 32]. Further large-scale study is needed to elucidate the association of glycemic control within the first two weeks from onset of COVID-19 with antibody response to SARS-CoV-2 and COVID-19 prognosis. A randomized clinical trial recently showed that an antibody cocktail (casirivimab and imdevimab) against SARS-CoV-2 spike protein reduced the risk of COVID-19 related hospitalization or death [33]. The findings in the present study potentially indicate that this type of antibody cocktail therapy may be particularly efficacious in patients with newly diagnosed diabetes, relative to all patients with diabetes.

There were several limitations in this study. First, as a result of the single-center design, this study had a relatively small sample size and homogeneous patient characteristics. In addition, because the date on which antibody measurements were performed varies from patient to patient, the estimated kinetics of the daily antibody response after the onset of disease shown in our study may not fully reflect an individual's antibody response. Thus, the generalizability of our findings may be limited. Second, owing to the wide range of SARS-CoV-2 assays, the IgG-S1 antibody tests used in this study may not have targeted the same spike protein domain as other studies. Third, we could not assess the neutralizing antibody response. Fourth, although propensity score matching (PSM) was utilized, unmeasured confounding variables could have affected the differences in antibody response between patients with diabetes and those without. Fifth, all patients diagnosed with newly diagnosed diabetes had an HbA1c ≥ 6.5%, however, considering that patients with COVID-19 may be temporarily hyperglycemic, patients who did not meet the diagnostic criteria for diabetes at the time of COVID-19 non-infection may be included among the newly diagnosed diabetes. Sixth, in Japan, two lineages (B.1.1.284 and B.1.1.214) were reported to be predominant between March 2020 and November 2020 [34]. Then, by about April 2021, B.1.1.214 were rapidly replaced by R.1 variant (B.1.1.316) and Alpha variant (B.1.1.7) [35]. Thus, although the patients included in our study might infect different variant, no identification of the infecting strain was made for each patient. It is still unknown whether the antibody response is different for each mutant strain, therefore, further studies examining whether patients with newly diagnosed diabetes have an impaired antibody response in each variant of SARS-CoV-2.

## Conclusion

In conclusion, this study demonstrated that patients with type 2 diabetes can produce IgG-N and IgG-S1 that are comparable to those in the general population, even after controlling for confounding variables with PSM. Furthermore, we discovered that IgG-N and IgG-S1 levels during the second week of the disease after symptom onset are significantly lower in patients with newly diagnosed diabetes than in those with previously diagnosed diabetes. This result presumably indicates that by ensuring that physicians test patients for undeclared diabetes when treating COVID-19, and by implementing strict glycemic control in patients with newly diagnosed diabetes, that early and sufficient antibody production may be ensured. This may in turn improve the prognosis of COVID-19 in this patient population.

## Data Availability

The datasets used and/or analyzed during the current study are available on reasonable request from the corresponding author.

## Abbreviations

SARS-CoV-2: Severe acute respiratory syndrome coronavirus 2
IgG: Immunoglobulin G
IgG-N: Immunoglobulin G antibodies specific for nucleocapsid protein
IgG-S1: Immunoglobulin G antibodies specific for the S1 subunit of the spike protein
COVID-19: Coronavirus disease 2019
PSM: Propensity score matching
HbA1c: Hemoglobin A1c
AU: Arbitrary units
BMI: Body mass index
COPD: Chronic obstructive pulmonary disease
CVD: Cardiovascular disease
IQR: Interquartile range
CRP: C-reactive protein
